# A Clinician’s Guide to Management of Intra-abdominal Hypertension and Abdominal Compartment Syndrome in Critically Ill Patients

**DOI:** 10.1186/s13054-020-2782-1

**Published:** 2020-03-24

**Authors:** Inneke E. De Laet, Manu L. N. G. Malbrain, Jan J. De Waele

**Affiliations:** 1 Intensive Care Unit and High Care Burn Unit, Ziekenhuis Netwerk Antwerpen, ZNA Stuivenberg, Antwerp, Belgium; 2 Department of Intensive Care Medicine, University Hospital Brussels (UZB), Jette, Belgium; 3 Faculty of Medicine and Pharmacy, Vrije Universiteit Brussel (VUB), Campus Jette, Jette, Belgium; 4 Department of Critical Care Medicine, Ghent University Hospital, Ghent, Belgium

## Abstract

This article is one of ten reviews selected from the Annual Update in Intensive Care and Emergency Medicine 2020. Other selected articles can be found online at https://www.biomedcentral.com/collections/annualupdate2020. Further information about the Annual Update in Intensive Care and Emergency Medicine is available from http://www.springer.com/series/8901.

## Introduction

Intra-abdominal hypertension (IAH) and abdominal compartment syndrome (ACS) are established causes of morbidity and mortality in critically ill patients [1]. When interest in postoperative IAH after major vascular, trauma, and general surgery arose in the 1980s, overt ACS was the only clinical syndrome recognized and decompressive laparotomy the only definitive treatment [2]. Since then, less extreme elevations in intra-abdominal pressure (IAP), defined as IAH, have been recognized to be highly prevalent among all types of patients admitted to the intensive care unit (ICU) [3].

Significant advances in the understanding of the pathophysiology, diagnosis, and management of IAH and ACS have occurred over the last few decades. The importance of IAH has been studied specifically in critically ill patients, leading to a better understanding of the mechanisms of organ dysfunction due to increased IAP and earlier opportunities for therapeutic intervention. Further, medical and minimally invasive techniques have been developed and reported to be potentially effective in small studies [4].

The World Society for the Abdominal Compartment Syndrome (WSACS, recently renamed as WSACS—the Abdominal Compartment Society [5]) was founded in 2004 to “promote research, foster education and improve the survival of patients with IAH/ACS.” Consensus papers on IAP measurement and diagnosis and management of IAH/ACS were first published in 2006 and 2007 [1, 6] and a medical management algorithm in 2009 [7]. Subsequently, in 2013, the WSACS published an updated evidence-based version of the definitions, guidelines, and medical management algorithm using GRADE methodology (Box 1) [8]. In this last manuscript, the definitions relating to IAP were updated.

**Box 1:** Definitions Related to Intra-abdominal Pressure (IAP) According to the World Society for the Abdominal Compartment Syndrome (WSACS) 2013 Guidelines (Adapted from [8] Under the Terms of the Creative Commons Attribution Noncommercial License)

No.	Definition
1.	IAP is the steady-state pressure concealed within the abdominal cavity
2.	The reference standard for intermittent IAP measurements is via the bladder with a maximal instillation volume of 25 ml of sterile saline
3.	IAP should be expressed in mmHg and measured at end expiration in the supine position after ensuring that abdominal muscle contractions are absent and with the transducer zeroed at the level of the midaxillary line
4.	IAP is approximately 5–7 mmHg in critically ill adults
5.	IAH is defined by a sustained or repeated pathological elevation in IAP ≥12 mmHg
6.	ACS is defined as a sustained IAP >20 mmHg (with or without an APP <60 mmHg) that is associated with new organ dysfunction/failure
7.	IAH is graded as follows:
	Grade I, IAP 12–15 mmHg
	Grade II, IAP 16–20 mmHg
	Grade III, IAP 21–25 mmHg
	Grade IV, IAP >25 mmHg
8.	Primary IAH or ACS is a condition associated with injury or disease in the abdominal pelvic region that frequently requires early surgical or interventional radiological intervention
9.	Secondary IAH or ACS refers to conditions that do not originate in the abdominopelvic region
10.	Recurrent IAH or ACS refers to the condition in which IAH or ACS redevelops following previous surgical or medical treatment of primary or secondary IAH or ACS
11.	APP = MAP – IAP
12.	A polycompartment syndrome is a condition where two or more anatomical compartments have elevated compartmental pressures
13.	Abdominal compliance is a measure of the ease of abdominal expansion, which is determined by the elasticity of the abdominal wall and diaphragm. It should be expressed as the change in intra-abdominal volume per change in IAP
14.	The open abdomen is one that requires a temporary abdominal closure due to the skin and fascia not being closed after laparotomy
15.	Lateralization of the abdominal wall is the phenomenon where the musculature and fascia of the abdominal wall, most exemplified by the rectus abdominis muscles and their enveloping fascia, move laterally away from the midline with time

*ACS* abdominal compartment syndrome, *MAP* mean arterial pressure, *IAH* intra-abdominal hypertension, *APP* abdominal perfusion pressure

**Fig. 1:**
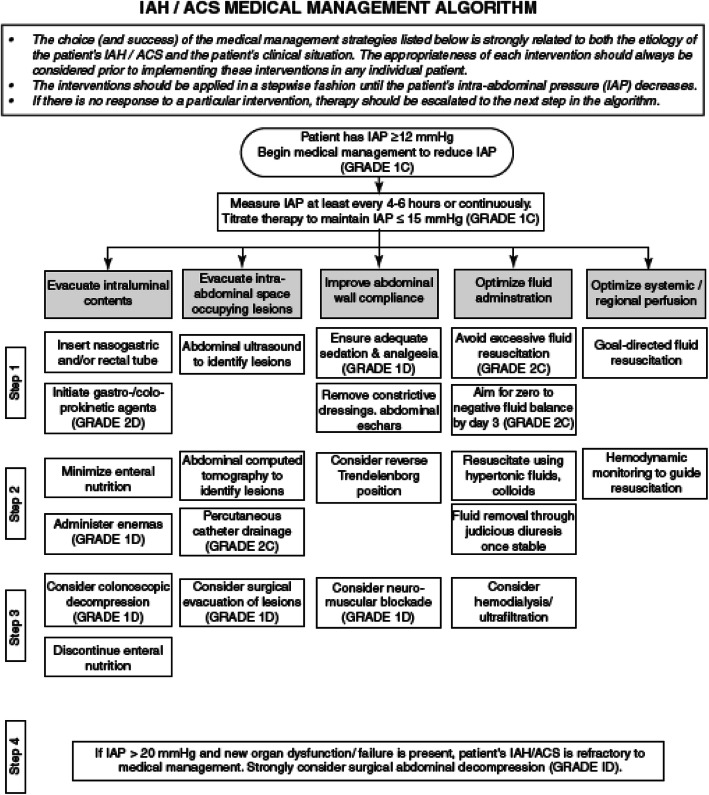
WSACS medical management algorithm as presented in the 2013 guidelines. *IAH* intra-abdominal hypertension, *ACS* abdominal compartment syndrome, *IAP* intra-abdominal pressure. Adapted from [8] under the terms of the Creative Commons Attribution Noncommercial License

The current medical management algorithm for IAH/ACS still has some limitations (Fig. 1). First, there is not enough evidence to support some of the interventions described in the algorithm. Second, the use of the algorithm at the bedside also requires an experienced clinician to select the treatment best suited to an individual patient as it does not provide clear, easy, patient-specific recommendations. Finally, management recommendations are chiefly based on a measured IAP value only, an approach likely to underestimate the importance of the dynamic evolution in the patient’s situation. Depending on the course of disease and concomitant organ dysfunction, some cases of ACS can be managed conservatively whereas some cases of IAH may require immediate aggressive treatment including fast decision to proceed to decompressive laparotomy before reaching the value of 20 mmHg of IAP. This is important because use of decompressive laparotomy is associated with a number of potential complications (e.g., massive ventral hernia, enteric fistulae, and intra-abdominal sepsis), increased morbidity, and decreased quality of life, especially in younger patients [9–12].

The philosophy of the WSACS Guidelines has been to publish the best available evidence at the time of writing, with the hope that future research would necessitate ongoing revisions and updating of the Guidelines. The aim of this chapter is to provide the reader with a conceptual framework of how to translate the principles of the formal Consensus Guidelines into a practical approach at the bedside to manage a specific patient with IAH and ACS, taking into account patient physiology, current scientific evidence, and clinical experience.

## Managing IAH and ACS: The Triangle Paradigm

**Fig. 2:**
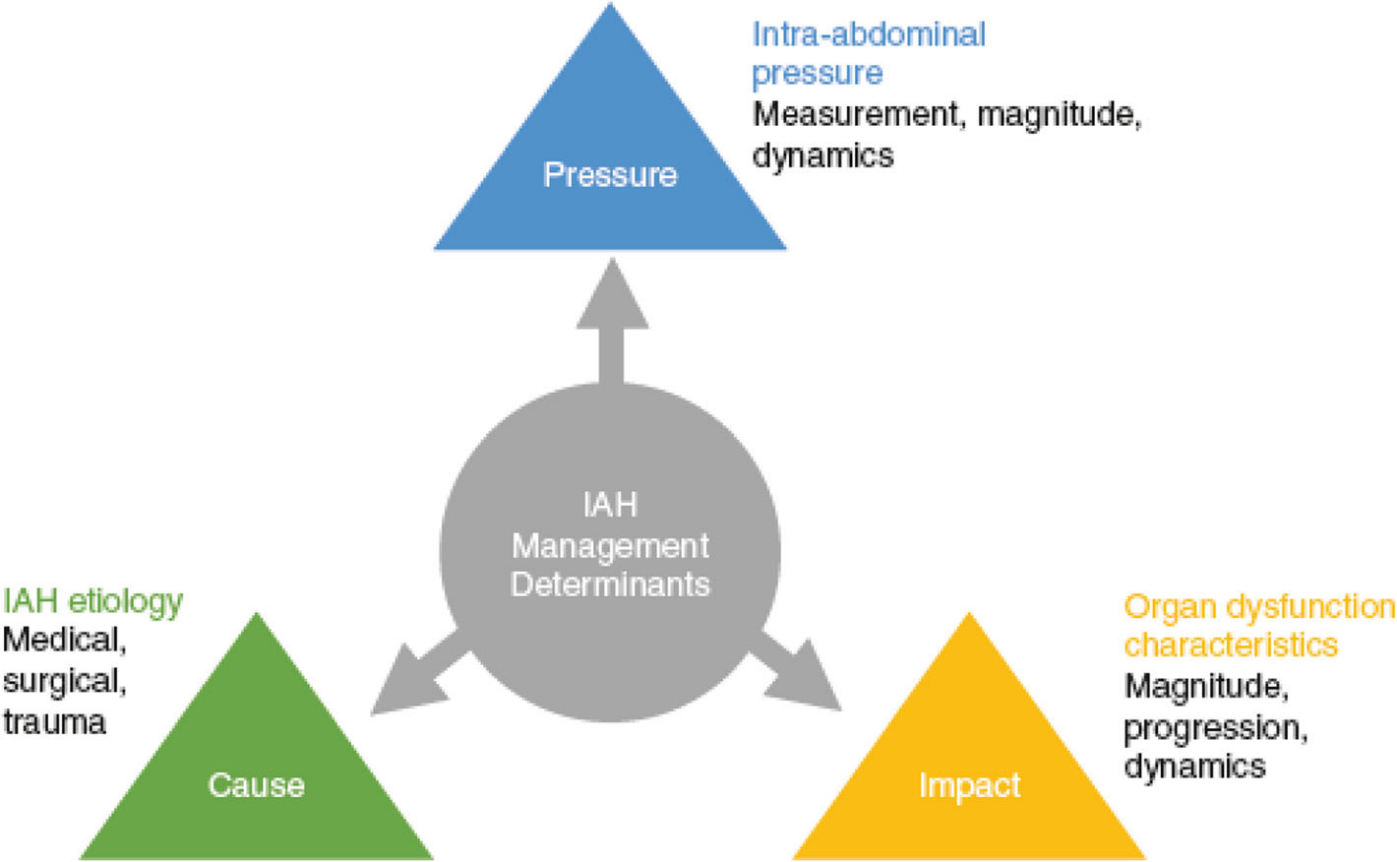
The triangle perspective on the management of intra-abdominal hypertension/abdominal compartment syndrome (IAH/ACS) in the individual patient

It is important to understand that IAH, in contrast to ACS, is a continuum from (often) asymptomatic elevation of IAP to an immediately life-threatening situation (fulminant ACS), where dynamic evolution in both directions is possible. Therefore, it is difficult to identify triggers for interventions that may lead to complications (e.g., percutaneous drainage) or have adverse effects (e.g., sedation, muscle relaxation). Despite this, the optimal treatment choice for a specific patient with IAH/ ACS should take into account three critical elements: (1) the measured IAP value (or the degree/magnitude of IAP increase); (2) organ dysfunction characteristics (or the impact of increased IAP); and (3) nature and course of the underlying disease (Fig. 2). Using this triangular treatment paradigm enables us to fully acknowledge the importance of the two other factors in addition to the measured IAP value.

### Intra-abdominal Pressure (Culprit)

Although the IAP value has always been considered the most important factor in managing IAH/ACS, it should always be viewed within its context. Factors that need to be considered in an individual patient include the IAP measurement strategy and the context in which IAP is measured, the expected baseline value of IAP, the evolution of IAP over time, and the duration of time that the patient has already been exposed to IAH.

#### IAP Measurement and Interpretation

The reference standard for intermittent IAP measurement is via the bladder with a maximal instillation volume of 25 ml of sterile saline. IAP should be measured at endexpiration in the supine position after ensuring that abdominal muscle contractions are absent and with the transducer zeroed at the level where the midaxillary line crosses the iliac crest [8]. This generally means that IAP measurement is most reliable in completely sedated, mechanically ventilated patients. However, many mechanically ventilated patients in the ICU are at some stage of a weaning process, exhibiting spontaneous breathing movements and possible patient-ventilator asynchrony and pain or distress. Similarly, critically ill patients who are not mechanically ventilated may be managed with noninvasive ventilation or exhibit respiratory failure, forced expiration, and pain or stress. All of the above processes may lead to abdominal wall muscle contraction and increased IAP that may not reflect an increase in intra-abdominal volume [13, 14]. Although there are no data as to whether increased IAP due to abdominal muscle activity in these groups of patients has the potential to cause organ dysfunction, it has been reported that in awake, non-critically ill patients without suspicion of IAH, IAP can be as high as 20 mmHg without causing discernible organ dysfunction [15]. The impact of high positive end-expiratory pressure (PEEP; >12 cmH_2_O) on IAP is considered to be mild and adds 1–2 mmHg at most [16]. As deepening of sedation or using neuromuscular blocking agents may help to decrease IAP and control IAH for a limited period of time, it needs to be considered that deepening of sedation may have deleterious effects on hemodynamics. Switching from assisted to controlled mechanical ventilation may sometimes result in a significant increase in intrathoracic pressure even with muscle relaxation and the expected positive effect on IAP will be negligible compared to its negative effects.

#### Baseline IAP Value and Dynamics

The baseline IAP may vary in individual patients. Obese patients in particular have higher baseline IAP values [17], which in some cases may be higher than the threshold for IAH. One review found that IAP in individuals with a normal weight was around 5–6 mmHg, whereas it was much higher in obese patients with values above 12 mmHg and even above 14 mmHg in morbid obesity [16]. Other conditions associated with “physiologically” increased IAP include pregnancy [18] and liver cirrhosis with ascites [19]. Although this chronic IAP elevation may contribute to chronic forms of organ failure, including chronic kidney failure in patients with congestive heart disease and obesity [20] or pseudotumor cerebri in patients with obesity [21], slight increases from a higher starting value may have limited implications in critically ill patients. As such, an IAP of 16 mmHg may be insignificant if the baseline value was 13 mmHg, where it may cause organ injury if the baseline value was 6 mmHg. Unfortunately, the baseline IAP value is usually unknown and this effect is difficult to quantify.

#### Duration of IAH

In situations where exposure to IAH has already been prolonged (e.g., several days, in cases of delayed IAH diagnosis), organ dysfunction may not be reversible as quickly or fully as in more acute situations. We hypothesize that interventions aimed at lowering IAP are unlikely to have an immediate beneficial effect on organ function in this context, especially when IAH has caused or contributed to cellular organ injury (e.g., acute tubular necrosis). This highlights the importance of IAP monitoring in at-risk patients to avoid delayed diagnosis [22]. On the other hand, one measurement of elevated IAP does not constitute a definite diagnosis of IAH/ACS (as highlighted by the definitions in Box 1). Repetitive measurements are more likely to ascertain true IAP values and unmask potential measurement errors. Mild elevation of IAP, measured at one time point, is unlikely to cause organ dysfunction and seldom warrant immediate intervention, but should lead to repeated IAP measurement.

### Organ (Dys)Function (Impact)

The second element of the triangle to consider is the resultant degree of organ dysfunction thought to be secondary to IAH and the rapidity with which it occurred. Many experimental studies have shown that subclinical organ injury develops at levels of IAP previously deemed to be safe (IAP between 12 and 15 mmHg), but as IAP increases, organ dysfunction will become more pronounced and a dose-dependent relationship between IAP and organ dysfunction has been demonstrated in many studies [23].

#### Severity of Organ Dysfunction

One of the key features of ACS is organ dysfunction and the absence of organ dysfunction should raise doubts about the reliability of the measurement or the interpretation of the IAP value. The most extreme and urgent form of organ dysfunction in patients with ACS is the inability to ventilate, which requires urgent action. Another very frequent form of IAH-induced organ dysfunction is IAH-induced acute kidney injury (AKI) [24]. There is extensive experimental evidence that AKI occurs at IAP levels as low as 12 mmHg [25]. In patients with ACS, AKI is usually firmly established with anuria and need for renal replacement therapy (RRT) unless early intervention is used to prevent this [25]. Organ dysfunction is not limited to the respiratory or renal system and may include hemodynamic instability, metabolic failure, gastrointestinal failure, and even intracranial hypertension [26]. Often multiple organ systems will fail, and the clinical picture can mimic many conditions (e.g., septic shock, hypovolemia) associated with multiple organ dysfunction syndrome (MODS). Compartment pressures can also be increased in more than one compartment and this has been referred to as the polycompartment syndrome [27].

#### Organ Dysfunction Duration and Dynamics

The speed at which organ function deteriorates and the time-dependent relationship with the increase in IAP are important elements to consider. A sudden increase in intra-abdominal volume, causing a sudden increase in IAP with subsequent organ dysfunction, warrants more aggressive treatment than a situation where a condition frequently associated with MODS is diagnosed concurrently with IAH and organ dysfunction. Indeed, in many conditions that are associated with IAH, the pathophysiology of the underlying disease (e.g., severe trauma, severe acute pancreatitis, or burns) may cause severe organ dysfunction and the exact role of increased IAP superimposed on this “primary” organ injury may be difficult to estimate. Baseline organ dysfunction (i.e., before IAH was present) as well as dynamics between concurrent increase in IAP and deterioration of organ function may offer a clue.

### Etiology of IAH/ACS (Cause)

The third element to consider in IAH management is the etiology of the elevated IAP, which allows selection of the best possible treatment option. The course of disease also needs to be considered. An initial increase in IAP up to 18 mmHg after elective abdominal hernia repair may be well tolerated [28] and could be just observed, whereas the same value of IAP in a patient with severe acute pancreatitis and shock still needing massive fluid resuscitation to preserve organ perfusion presents a high risk for developing ACS and needs immediate attention and measures (e.g., sedation, muscle relaxation) to control the IAP.

All reasonable attempts should be made to ascertain the underlying disease leading to elevated IAP before starting treatment. Knowledge of the patient’s medical history and present condition and a full general and abdominal clinical examination usually offer the first clues. Directed imaging, such as ultrasound or computed tomography (CT), may also be necessary. A plethora of risk factors for IAH/ACS has been described, but they can be largely divided into three categories: increased intra-abdominal volume, decreased abdominal compliance, and a combination of both [13].

#### Increased Intra-abdominal Volume

This can be caused by increased intraluminal or extraluminal volume within the abdominal cavity. The presence of increased intraluminal volume can be suspected based on the clinical circumstances and diagnosed with medical imaging techniques if indicated (e.g., gastric distention after gastroscopy due to gas insufflation, added colonic volume in *Clostridium difficile* colitis [29], or severe constipation). Increased extraluminal volume may accumulate freely in the abdominal cavity or localized in abdominal collections. Free abdominal air, fluid, or blood can be diagnosed easily by bedside ultrasound and can be evacuated by percutaneous catheter drainage. Extraluminal abdominal collections are mostly associated with underlying abdominal diseases (e.g., pancreatitis, abdominal sepsis, or abdominal hematoma) and usually require abdominal ultrasound or CT imaging for accurate diagnosis and treatment. Tissue edema—often in a context of resuscitation or fluid overload—may be another cause of increased extraluminal volume, without any discernible collections. In rare cases, IAH/ACS may be caused by increased native solid organ volume (e.g., splenomegaly or in solid organ transplants [30], e.g., in children receiving adult organs [31]).

#### Decreased Abdominal Wall Compliance

Abdominal wall compliance is a measure of the ease of abdominal expansion, which is determined by the elasticity of the abdominal wall and diaphragm [32]. When abdominal wall compliance is decreased, any increase in intra-abdominal volume is much more likely to produce a significant increase in IAP. Risk factors for decreased abdominal wall compliance can be divided into three categories, including those related to (1) body anthropomorphism and habitus (e.g., age, morbid obesity); (2) abdominal wall (e.g., burn eschars, rectus sheath hematoma, tight sutures or bandages, ventral hernia repair, prone positioning); and (3) comorbidities (e.g., capillary leak due to sepsis, burns, trauma, or pancreatitis) [33]. Large-volume fluid resuscitation, usually related to systemic inflammatory syndrome and biomediator activation, is one of the most important risk factors for the development of IAH/ ACS, due to its combined effects of increased intra-abdominal volume (both intraand extraluminal due to ascites formation, gut edema, and ileus) and decreased abdominal wall compliance due to tissue edema of the abdominal wall. Respiratory cycle-related variations in IAP have been found to linearly increase with end-expiratory IAP and reflect abdominal wall compliance [34].

## A Practical Approach Based on the IAH Triangle

**Fig. 3:**
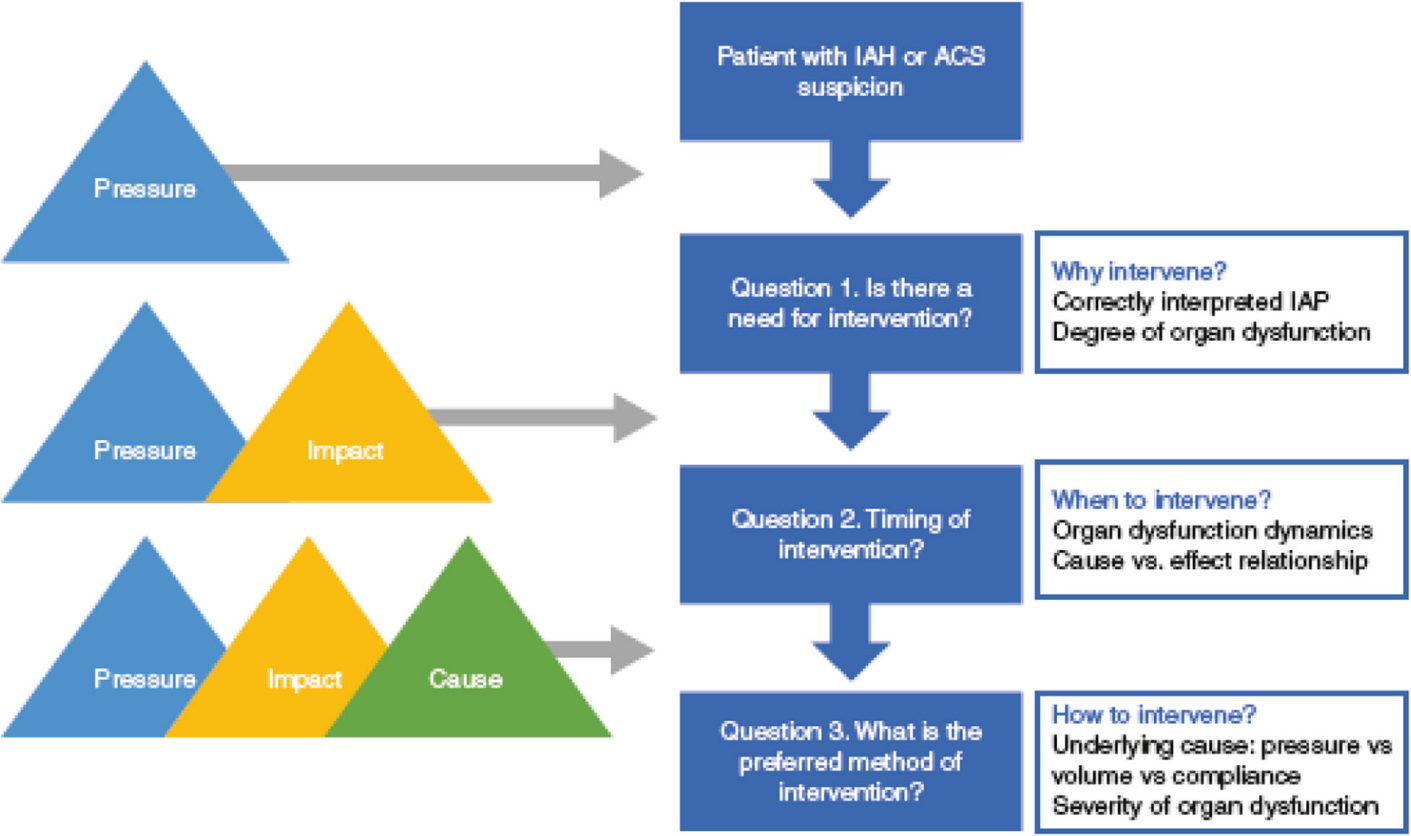
Elements to be considered in decision-making for management of intra-abdominal hypertension (IAH). *ACS* abdominal compartment syndrome, *IAP* intra-abdominal pressure

The first two elements of the triangle (pressure and impact) will determine whether or not active attempts to decrease IAP should be considered, in what timeframe these attempts should produce a clinically relevant result, and what level or invasiveness (and possibility of complications) is required. The third element (cause) will determine which treatment option will most likely produce the desired result. At the bedside, three critical questions should be asked once IAH/ACS has been diagnosed (Fig. 3).

### Is an Intervention Required?

Why intervene: The decision to intervene will be guided by the presence of organ dysfunction caused by a relevant increase in IAP in a patient who has been diagnosed with a condition that may be associated with IAH and in which an intervention is expected to have a beneficial impact on IAP as well as on organ function. The IAP value, the evolution of IAP over time, and the degree of organ dysfunction are the most important considerations. However, the measured IAP value should be interpreted carefully. If IAP is elevated in semiconscious or fully awake patients and organ function is normal or improving, techniques to reduce IAP are probably less warranted and may cause unnecessary complications. If IAP is normal after analgesia/sedation, IAH is unlikely to be a contributing factor to organ dysfunction. If IAP remains increased, an underlying cause of IAH is likely and additional diagnostic and/or therapeutic interventions are warranted.

### How Urgent Is the Effect of the Intervention Required?

When to intervene: The urgency of an intervention in the setting of IAH/ACS depends on the measured IAP value, the rate of IAP increase, and the degree of organ dysfunction. In most situations, starting stepwise management should not be delayed and some situations require immediate invasive intervention. In general, in patients with primary ACS, intervention is more urgent than in patients with secondary ACS where the clinician has more time to intervene. If adequate oxygenation and/or ventilation cannot be maintained despite optimal ventilator settings, or circulation is severely compromised despite adequate fluid resuscitation and vasopressor support, immediate decompression may be required—irrespective of the other interventions. If organ function is slowly deteriorating along with a gradually increasing IAP, using a technique expected to have a slower effect on IAP may be considered, if the potential for serious complications can be avoided by this strategy.

### What Is the Best Method of Intervention?

How to intervene: The method of choice for treating IAH will be guided by both the cause that led to the IAH and the degree of organ dysfunction. Knowing the cause of IAH can help predict the effect of a specific intervention on IAP, both in magnitude and time to effect. The degree and dynamics of organ dysfunction should be considered to determine the desired decrease in IAP and the time allowed to achieve it. Many techniques to decrease IAP have been described and interventions may be aimed at lowering intra-abdominal volume (intra- or extraluminal volume), improving abdominal compliance, or both.

#### Reducing Intraluminal Volume

Evacuation of excess volume from the gastrointestinal tract can be accomplished by prokinetics and/or enemas. Decompression of the gastrointestinal tract by nasogastric and/or rectal tubes or endoscopic decompression can be performed quickly and safely, but only the most proximal and distal parts of the gastrointestinal tract are accessible for easy intervention [8], thereby limiting their expected effectiveness in some patients. IAH/ACS due to small bowel dilatation may be difficult to treat noninvasively. Even if surgery is not required for treatment of the underlying condition, decompressive laparotomy may be necessary, especially as the combination of abdominal visceral edema and increased IAP poses a significant risk for bacterial translocation or even bowel ischemia [35].

#### Reducing Extraluminal Volume

Percutaneous catheter drainage can be used as a definitive treatment in some cases (e.g., ascites in liver cirrhosis [36], burn patients with ACS [37]), but can also be used as a temporary measure in cases where investigation of the underlying disease is ongoing but organ dysfunction requires urgent decompression (e.g., decompression of pneumoperitoneum before evaluation for gastrointestinal tract perforation [38]) or after definitive treatment of the underlying condition to treat any residual IAH/ACS (e.g., evacuation of free abdominal blood after endovascular aortic reconstruction for ruptured aortic aneurysm). This is a direct challenge to the classical adage that a diagnosis of overt ACS equals the need for decompressive laparotomy while, even in extreme circumstances, the etiology of ACS should be considered. As an example, several cases of ACS due to acute massive pneumoperitoneum, successfully treated with needle decompression, have been published [38].

#### Improving Abdominal Wall Compliance

Some conditions associated with impaired abdominal wall compliance can be easily corrected and enable fast and significant decrease in IAP. Burn eschars can be treated with escharotomy [39], tight bandages can be released, and body position can be changed [13]. For other causes of decreased abdominal wall compliance, fast release is not possible or not desirable (e.g., release of a tight hernia repair). In these cases, other techniques to improve abdominal wall compliance can be attempted (such as analgesia and/or sedation [40], neuromuscular blockers [41], and changing body position [42]) when indicated. Since small changes in intra-abdominal volumes can lead to significant changes in IAP in patients with decreased abdominal wall compliance, bedside ultrasound and removal of moderate amounts of ascites may offer relief of IAH/ACS, even if the main etiology of IAH is decreased abdominal wall compliance not amenable to nonsurgical treatment.

#### Decompressive Laparotomy

Decompressive laparotomy will decrease intra-abdominal volume in relation to the abdominal cavity and abdominal wall compliance and is as such the ultimate treatment for ACS. However, the consequences are considerable and even with improved open abdomen management techniques this should—based on current knowledge—only be reserved for treatment failures [10–12]. However, treatment failures should be identified swiftly when they occur and both the decision to proceed to decompressive laparotomy and the execution of that decision should not be delayed if the patient’s condition warrants urgent intervention. The anesthesiologist and/or intensivist should be aware that decompressive laparotomy can be a severe ischemia-reperfusion event, especially when IAP has been elevated for some time, and patients may require supportive measures to tolerate the intervention. After decompressive laparotomy, patients should still be treated according to the medical management principles, especially in terms of controlling fluid balance and improving abdominal compliance, in order to facilitate primary fascial closure. The success of this approach has been demonstrated by Cheatham et al. [43]. IAP should be monitored closely after decompressive laparotomy in order to prevent recurrent ACS [44].

## Supportive Management of the Patient with IAH/ACS

This chapter focuses on the treatment of IAH/ACS in terms of treatment aimed at reducing IAP. It is important to realize that the presence of IAH/ACS may lead to changes in general ICU management [45]. Respiratory management is affected since studies have shown that higher ventilation pressures (both PEEP and plateau pressures) can be used safely in patients with increased IAP and may be warranted in order to maintain alveolar recruitment [46]. Elevated IAP has profound effects on the cardiovascular system and the microcirculation; it changes normal values for hemodynamic monitoring and can mimic a state of fluid responsiveness [47]. Administration of a fluid bolus may temporarily improve tissue perfusion although fluid resuscitation is a major risk factor for (progression of) IAH/ACS [48]. Since IAH/ACS can have an impact on practically all organ systems, it should be a consideration in all aspects of supportive ICU management [49], although a complete discussion on this topic is beyond the scope of this manuscript. Secondary IAH/ ACS is mainly an iatrogenic disease related to fluid overload after resuscitation; therefore, a more restrictive fluid management approach with limitation of fluid intake or fluid removal with diuretics or RRT with net ultrafiltration may have a beneficial effect on outcomes [50].

## Conclusion

In 2013, the WSACS published evidence-based guidelines on the definitions, diagnosis, and treatment of IAH and ACS. Even with the implementation of these guidelines, making bedside decisions regarding the management of individual patients with IAH/ACS remains difficult, because of the wide variety of conditions associated with IAH/ACS, the broad spectrum of associated organ dysfunction, and the large number of treatment options available to decrease IAP. In this chapter, we provide a clinical framework that provides insight into how to use the guidelines when managing a specific patient in daily practice. The key message is that treatment should not be based solely on the degree of IAH, but also on the severity and dynamics of organ dysfunction as well as the etiology of IAH/ACS.

In general, the higher the IAP, the faster and more pronounced the rise in IAP and the more severe or deteriorating the organ dysfunction, the prompter and more aggressive treatment of IAH that is warranted. Therefore, frequent re-evaluation, taking into account the progression of IAH and course of disease and organ dysfunction, is necessary. If the underlying cause is well controlled and general condition is improving, the further course of IAH can usually be observed before initiating aggressive treatment. If there is underlying ongoing inflammation and fluid resuscitation continues, it is unlikely that IAH will decrease and more aggressive measures should be considered early.

## Data Availability

Not applicable.
